# Epigenetic, transcriptional, and functional characterization of myeloid cells in familial Mediterranean fever

**DOI:** 10.1016/j.isci.2024.109356

**Published:** 2024-02-29

**Authors:** Rutger J. Röring, Wenchao Li, Ruiqi Liu, Mariolina Bruno, Bowen Zhang, Priya A. Debisarun, Orsolya Gaal, Medeea Badii, Viola Klück, Simone J.C.F.M. Moorlag, Frank van de Veerdonk, Yang Li, Leo A.B. Joosten, Mihai G. Netea

**Affiliations:** 1 Department of Internal Medicine and Radboud Center for Infectious Diseases, Radboud university medical center, Nijmegen, the Netherlands; 2 Department of Computational Biology for Individualised Medicine, Centre for Individualised Infection Medicine (CiiM), a joint venture between the Helmholtz-Centre for Infection Research (HZI) and Hannover Medical School (MHH), Hannover, Germany; 3 TWINCORE, a joint venture between the Helmholtz-Centre for Infection Research (HZI) and Hannover Medical School (MHH), Hannover, Germany; 4 State Key Laboratory of Earth Surface Process and Resource Ecology and Ministry of Education Key Laboratory for Biodiversity Science and Ecological Engineering, College of Life Sciences, Beijing Normal University, Beijing 100875, China; 5 Department of Medical Genetics, Iuliu Haţieganu University of Medicine and Pharmacy, Cluj- Napoca, Romania; 6 Department of Immunology and Metabolism, Life and Medical Sciences (LIMES) Institute, University of Bonn, Bonn, Germany

**Keywords:** Health sciences, Immunology, Immune response

## Abstract

Familial Mediterranean fever (FMF) is a periodic fever syndrome caused by variation in *MEFV*. FMF is known for IL-1β dysregulation, but the innate immune landscape of this disease has not been comprehensively described. Therefore, we studied circulating inflammatory proteins, and the function of monocytes and (albeit less extensively) neutrophils in treated FMF patients in remission. We found that monocyte IL-1β and IL-6 production was enhanced upon stimulation, in concordance with alterations in the plasma inflammatory proteome. We did not observe changes in neutrophil functional assays. Subtle differences in chromatin accessibility and transcriptomics in our small patient cohort further argued for monocyte dysregulation. Together, these observations suggest that the *MEFV*-mutation-mediated primary immune dysregulation in monocytes leads to chronic inflammation that is subsequently associated with counterregulatory epigenetic/transcriptional changes reminiscent of tolerance. These data increase our understanding of the innate immune changes in FMF, aiding future management of chronic inflammation in these patients.

## Highlights

• We characterized innate immune function and circulating inflammatory proteins in FMF
• FMF monocytes display dysregulated cytokine and transcriptomic/epigenetic profiles
• We observed no changes in neutrophil IL-8, lactate, or ROS production
• Follow-up studies should improve on sample size and cohort characteristics

## Graphical abstract

**Figure:**
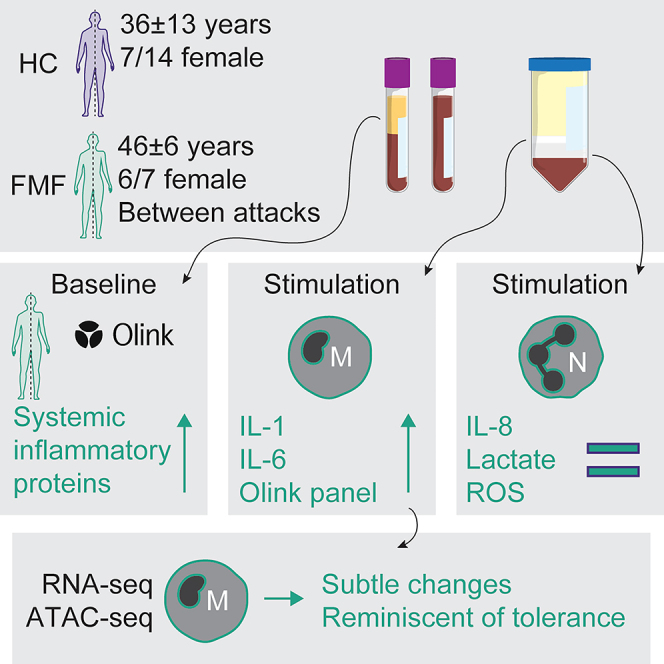

## Introduction

Familial Mediterranean fever (FMF) is the prototypical hereditary systemic autoinflammatory disease. Clinically characterized by recurrent but self-limiting bouts of fever and serositis,^1^ symptoms include heightened temperature (which may be sub-febrile), abdominal pain, chest pain, joint pain, and erysipelas-like lesions.^2^

The underlying cause of FMF are genetic mutations in the pyrin-encoding *MEFV* gene,^3^^,^^4^^,^^5^ with clear correlations between different known genotypes and disease severity.^6^^,^^7^^,^^8^^,^^9^^,^^10^^,^^11^ In the absence of disease-causing mutations in *MEFV*, pyrin acts as a sensor for inactivation of RhoA GTPases, a signal of pathogen activity.^12^^,^^13^ Upon activation, pyrin forms an inflammasome and enables canonical cleavage of pro-IL-1β and pro-IL-18 into their bioactive forms.^14^^,^^15^^,^^16^^,^^17^ In FMF, the threshold for activation of the pyrin inflammasome is decreased.^18^ If left untreated, this opens the way for symptomatic inflammatory episodes, to the great detriment of patients.

Several investigative efforts have previously been conducted to elucidate the immunological mechanisms of FMF. Overall, it seems that pro-inflammatory members of the IL-1 family (with the exception of IL-1 receptor antagonist),^11^^,^^19^^,^^20^^,^^21^ IL-6,^19^^,^^22^^,^^23^ IL-8,^24^ and (although there is no consensus) TNF^21^^,^^22^^,^^25^^,^^26^ are important mediators of inflammation in FMF. Together with the myeloid-restricted expression of pyrin,^27^ this points toward monocytes, macrophages, and neutrophils as the main cell populations mediating FMF. However, a comprehensive description of the inflammatory landscape of these cells is missing.

Another open question in the FMF literature is whether the myeloid cells in these patients have epigenetic and metabolic traits reminiscent of trained immunity. This recently described phenomenon is mediated via epigenetic and concomitant metabolic reprogramming of innate immune cells and their progenitors in the bone marrow.^28^ Interleukin-1 family members that are more strongly activated in FMF, such as IL-1β and IL-18 receptor, play a central role in trained immunity responses and induction.^29^ It has never been assessed if the IL-1-enrichment in FMF patients affects the epigenetic programming of monocytes specifically, although a recent study found that global DNA methylation is correlated with disease severity.^30^ This indicates a more detailed epigenetic investigation is warranted in FMF.

Here, we comprehensively studied the phenotype and functional responsiveness of monocytes and neutrophils in FMF patients that underwent treatment and were between inflammatory episodes. We report increased monocyte production of IL-1 and IL-6, concomitant upregulation of circulating inflammatory biomarkers, and a dysregulated monocyte program at the transcriptomic- and epigenetic levels.

## Results

### Study design and participant characteristics

**Figure 1:**
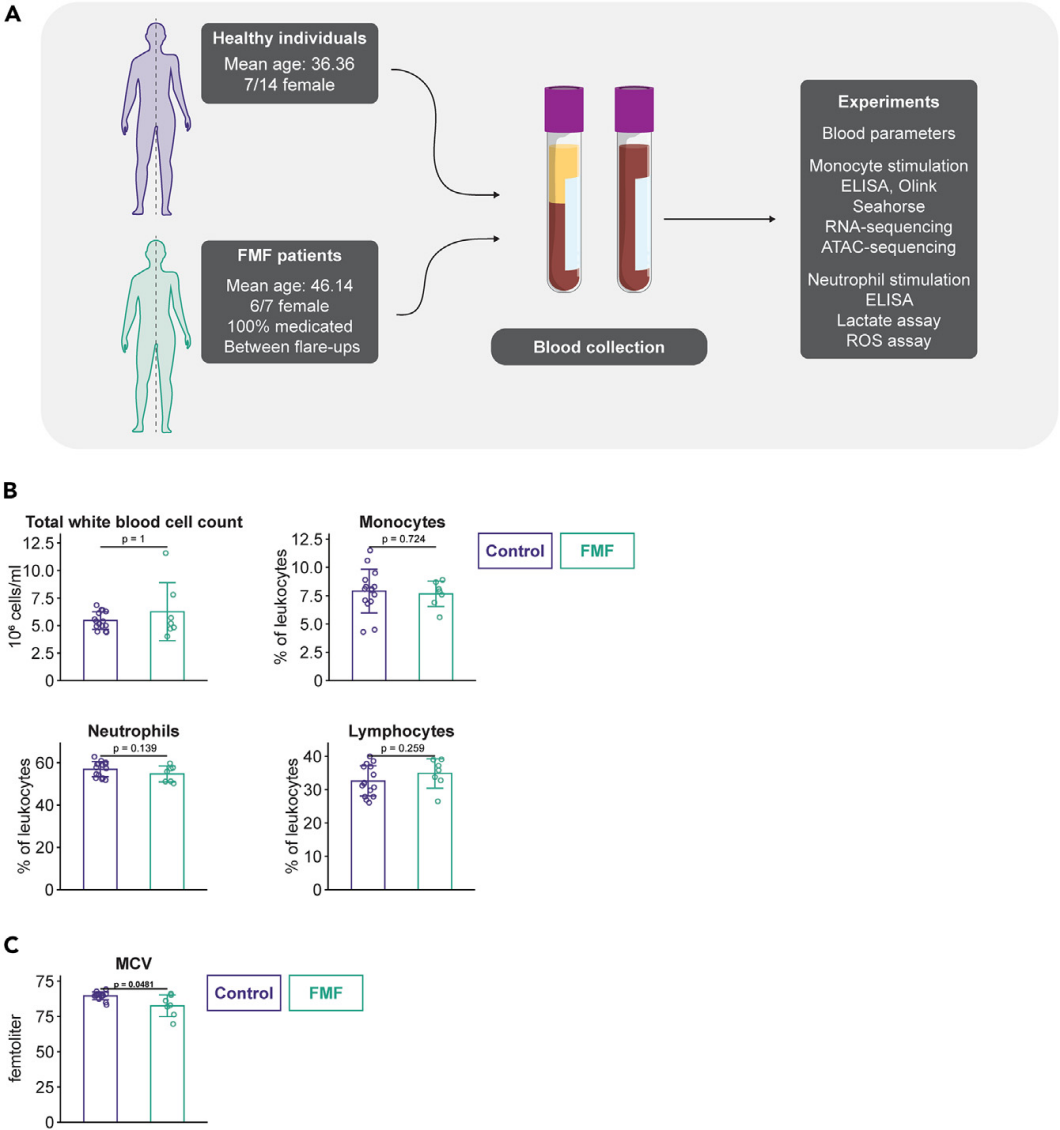
Study design and hematological parameters (A) Schematic overview of the study and participants. (B) Global white blood cell parameters. The total white blood cell counts are given as million cells per mL, whereas the proportions of major leukocyte populations are presented as percentages. (C) The mean corpuscular volume of red blood cells of the participants. Error bars indicate mean ± SD; each dot represents an individual participant (biological replicates).

Blood was collected from 7 patients with FMF and 14 healthy volunteers after informed consent. The study outline and participant are detailed in Figure 1A and Table S1, respectively. At the time of blood collection, the patients were treated with anti-inflammatory medication and were in a period of disease remission. With regards to the medication: all but one patient received colchicine. One patient received the TNF decoy receptor etanercept. Two patients received IL-1 blocking therapies (anti-IL-1β, canakinumab; or recombinant IL-1RA, anakinra). All but one of the patients used painkillers that are available over the counter (in the Netherlands), such as diclofenac or acetaminophen. Four of the patients also used prescription painkillers (oxycodone, a single patient used tramadol). The time since the last disease attack varied between patients (between 1 and 12 months, with the exception of 1 patient who had experienced an attack within the last month). In most patients, CRP was below 3 mg/mL (one patient had 6 mg/mL and the patient who had recently had a disease bout was at 12 mg/mL). The following *MEFV*-mutations were present in this study: p.M694I/p.R42W, p.M694V/p.M649V, homozygous p.M694V, p.M694I/p.R761H, p.M694V/p.V726A, p.M680I/p.E148Q (2 patients).

First, we measured immune cell populations in patients and controls: there were neither differences in the number of leukocytes, nor in the fraction of major immune cell subpopulations (Figure 1B). However, the mean corpuscular volume (MCV) of FMF patients was slightly but significantly lower compared to healthy individuals (Figure 1C), probably due to the chronic inflammation in these patients.

### Increased IL-6 and IL-1β production in FMF patients

**Figure 2:**
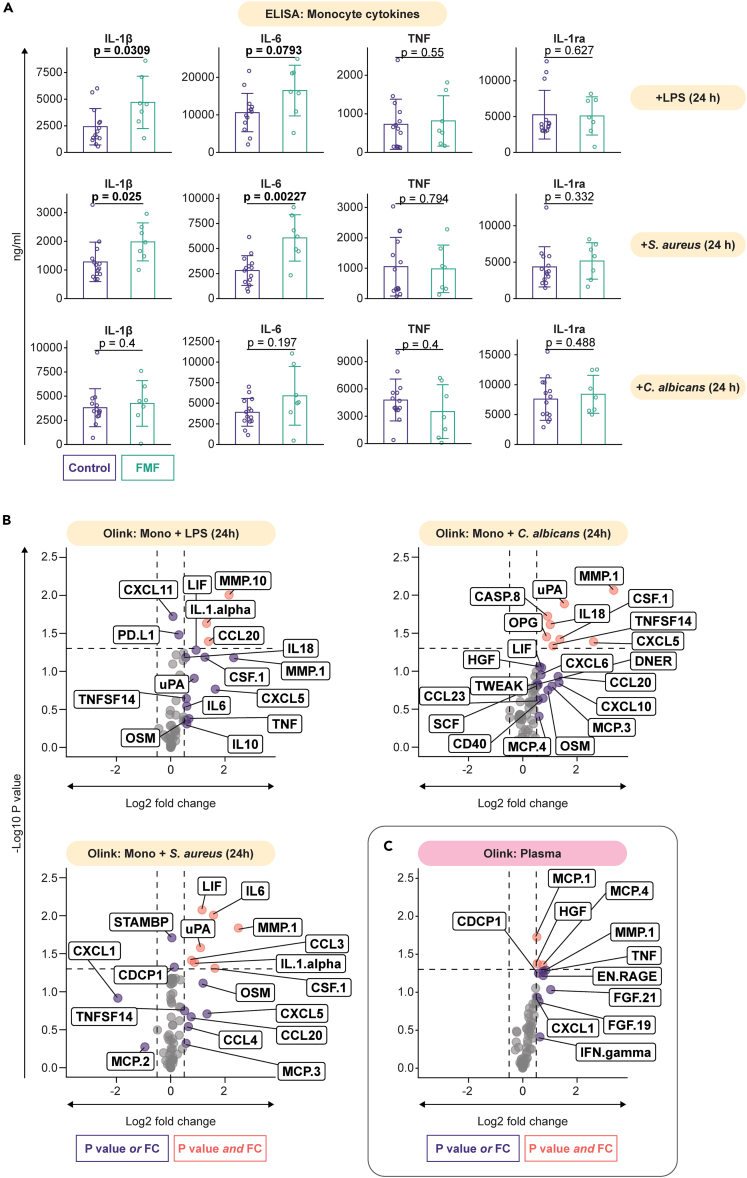
Monocyte cytokine responses and plasma inflammatory proteome (A) Cytokine responses (specifically IL-1β, IL-6, TNF, IL-1ra) of monocytes stimulated with LPS, heat-killed *S. aureus*, heat-killed *C. albicans*; measured by ELISA. (B) Inflammatory protein production by monocytes following stimulation with LPS, heat-killed *S. aureus*, heat-killed *C. albicans*; measured by Olink proximity extension assay (fold change represents FMF patients over healthy individuals). (C) Circulating inflammatory proteome of FMF patients over healthy individuals; measured by Olink proximity extension assay. Bar charts with error bars indicate mean ± SD, with each dot representing an individual participant (biological replicates).

Second, we sought to investigate the monocyte cytokine response toward various stimuli. We purified monocytes by negative selection using magnetic beads, and subsequently stimulated them for 24 h with microbial ligands (LPS, Pam_3_Cys), heat-killed whole pathogens (*Staphylococcus aureus*, *Candida albicans*, and *Aspergillus fumigatus*), or triggers of monocyte responses in the context of gout-related sterile inflammation (C16 alone or in combination with monosodium urate crystals (MSU)). We measured by ELISA the IL-1β, IL-6, TNF, and IL-1ra production in the supernatants (Figure 2A and S1A). As expected, there was a pattern of upregulated IL-1β production which reached statistical significance upon stimulation with LPS and *S. aureus*. Production of IL-6 was even more strongly upregulated, especially following stimulation with LPS, *S. aureus*, and Pam_3_Cys. On the other hand, production of TNF was not changed in FMF patients. The anti-inflammatory IL-1ra was upregulated in FMF patients in the RPMI control and upon stimulation with *Aspergillus* or C16 alone. Together, these data indicate that especially the production of monocyte-derived IL-1β and IL-6 is upregulated in FMF.

Third, to follow up on these findings, we performed a targeted proteomics analysis (Proximity extension assays platform, PEA-Olink) on the same cell culture supernatants (Figures 2B and S1B). To validate our results, we first compared the proteomics results against our previous ELISA analysis. While IL-1β is not part of the Olink inflammation panel, we found that production of IL-1α (which shares its genomic location with IL-1β) was increased in FMF patients upon stimulation with LPS, *S. aureus*, C16 alone, and Pam_3_Cys. These are the same stimuli that elicited the most pronounced difference in IL-1β responses. IL-6 production was also upregulated in Olink proteomics analyses after stimulation with LPS, Pam_3_Cys, C16 alone, *Aspergillus*, and most pronounced following *S. aureus*. TNF appeared weakly increased after LPS or *Aspergillus* but, in concordance with ELISA data*,* it was strongly upregulated in FMF patients following Pam_3_Cys. These results indicate that the ELISA and Olink proteomics analyses are mostly in agreement.

In addition, colony-stimulating factor 1 (CSF-1, also known as M-CSF) production was upregulated in FMF patients across all stimuli. The chemokines CXCL5, MCP-1, and MCP-4 were also broadly upregulated, while the matrix metalloproteinases MMP-1 and MMP-10 showed a similar pattern. Interestingly, in contrast to the cytokines, these proteins were also upregulated in the RPMI control samples, suggesting that these could be upregulated systemically between FMF flare-ups, even before an insult occurs.

### Systemic inflammation is increased in FMF patients outside inflammatory episodes

To investigate the systemic inflammatory status of these patients, we assessed the concentrations of inflammatory proteins in the plasma (Figure 2C). Using Olink proteomics analysis, we found that (in concordance with the results from stimulated monocytes) MMP-1, HGF, MCP-1, and MCP-4 were increased. Considering the whole inflammation panel, there seemed to be a broad trend toward upregulation in the plasma inflammatory proteome. These data reinforce the notion that patients with FMF experience a chronic low-grade inflammation even between attacks.^31^

### Neutrophil responses are not increased in FMF during disease remission

**Figure 3:**
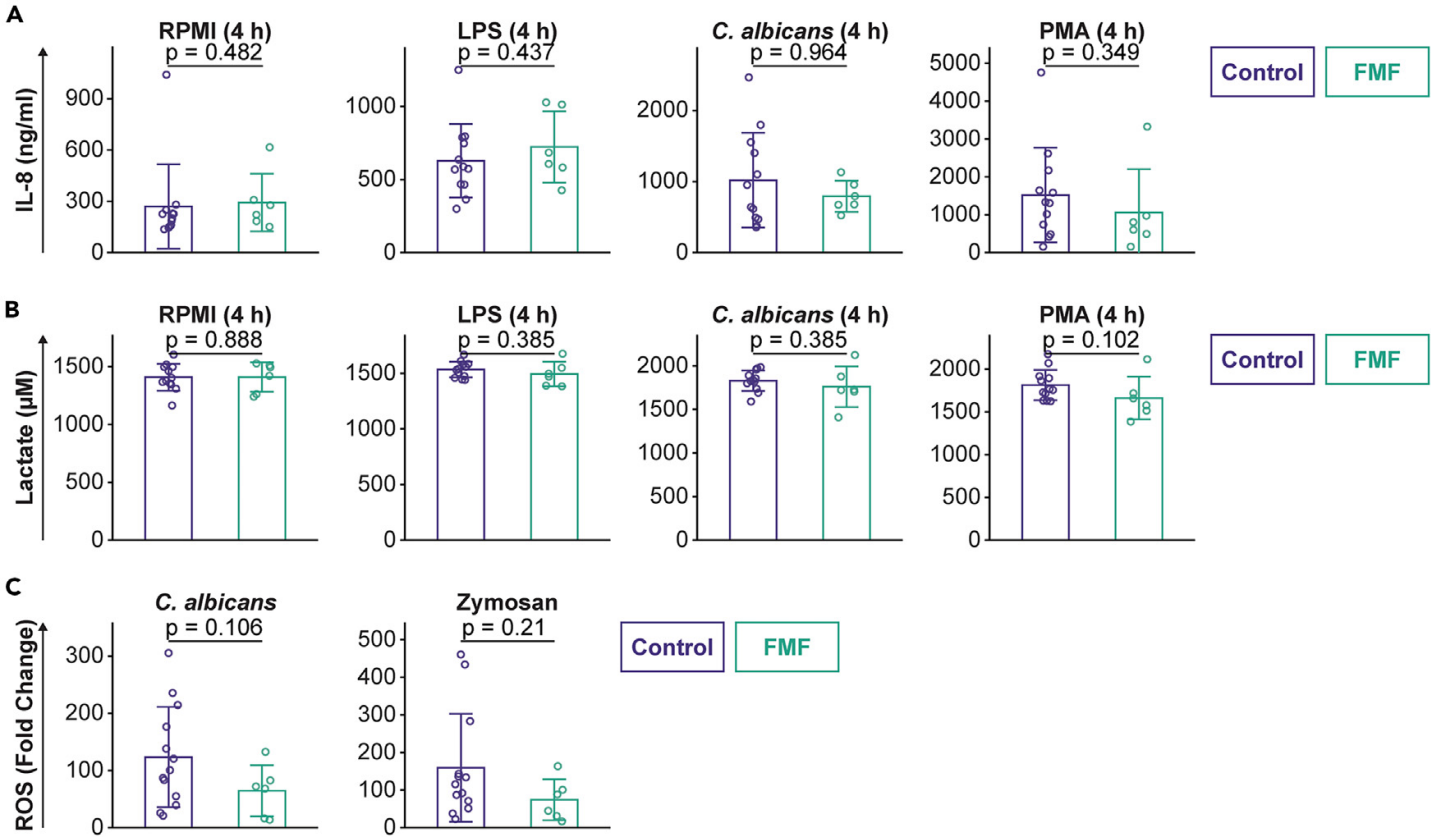
Neutrophil responses in FMF (A) Production of IL-8 by neutrophils, following stimulation with RPMI (as a control), LPS, heat-killed *C. albicans*, or PMA. (B) Production of lactate by neutrophils, following stimulation with RPMI (as a control), LPS, heat-killed *C. albicans*, or PMA. (C) Reactive oxygen species responses of neutrophils, following stimulation with heat-killed *C. albicans* or Zymosan particles. The fold-increase in ROS production was calculated over the baseline for each individual. Error bars indicate mean ± SD; each dot represents an individual participant (biological replicates).

We explored the functional responses of neutrophils in FMF patients compared to controls. Nota bene: neutrophil isolation failed for 1 healthy individual and we thus included only 13 controls for neutrophil analyses. The purified neutrophils were stimulated with LPS, *Candida*, or phorbol myristate acetate (PMA) for 4 h and thereafter inflammatory parameters were measured in the supernatants. While all stimulations elicited higher production of IL-8 compared to RPMI (Figure 3A), there were no differences between FMF patients and controls. Probing neutrophil metabolism, we measured lactate in the supernatants as a broad measure of potentially increased cellular activation. There were no differences between FMF patients and controls in lactate production (Figure 3B). Thereafter we examined another important function of neutrophils, which is the production of reactive oxygen species (ROS). We stimulated neutrophils with *Candida* or serum opsonized Zymosan particles and quantified the fold change of ROS production over RPMI-treated control cells. The upregulation of ROS production was considerable and particularly pronounced in neutrophils of healthy individuals. Although there was less strong induction of ROS generation in FMF patients, this was not statistically significant. Together, these data indicate that prototypical functions of neutrophils are not increased in this set of FMF patients between attacks, compared to healthy subjects.

### Monocyte metabolism does not drive their increased responses

**Figure 4:**
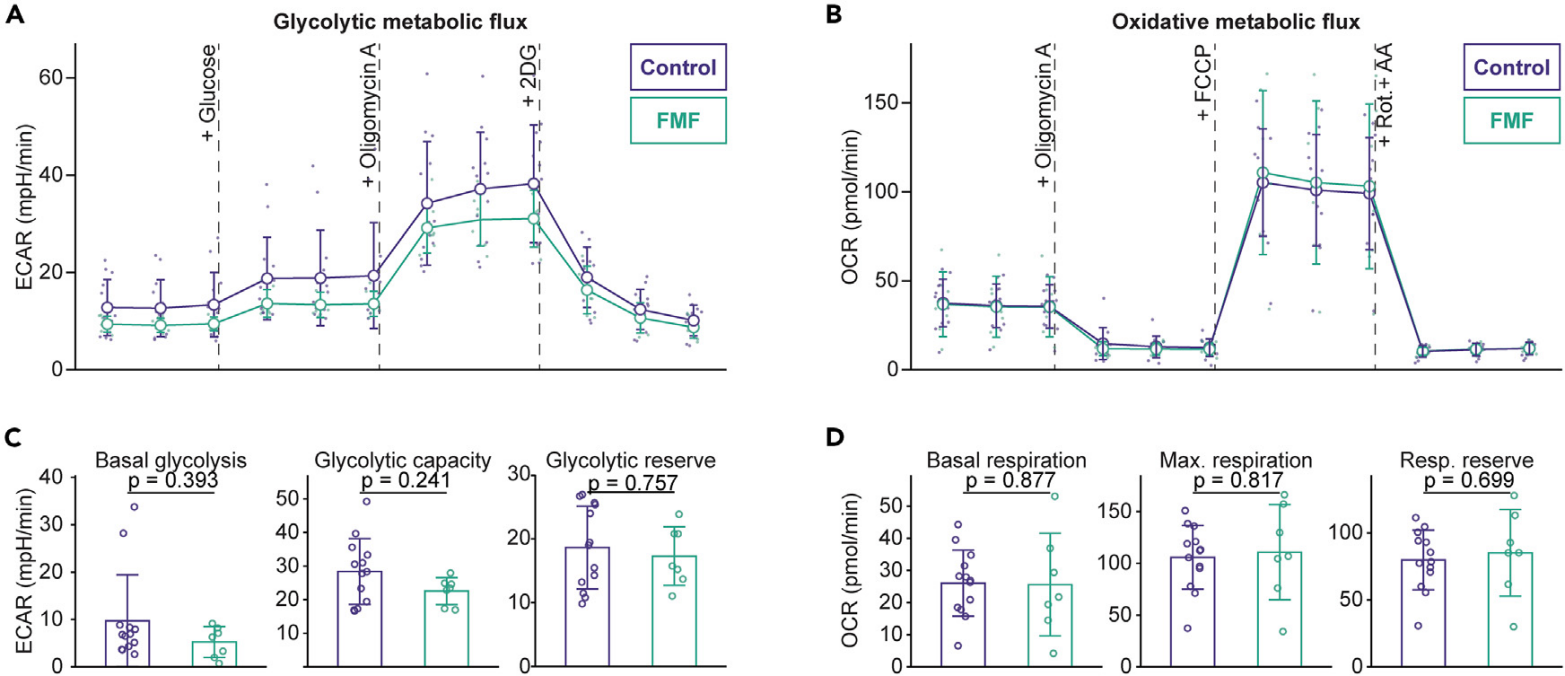
Metabolic flux analyses of monocytes (A) Global overview of extracellular acidification rate (a proxy for glycolysis) during glycolysis stress-testing. (B) Global overview of oxygen consumption (a proxy for oxidative phosphorylation) during mitochondrial stress-testing. (C) Detailed glycolytic parameters. (D) Detailed respiratory parameters. For panels A and B: dot and error bars represent mean and SD, respectively. The small points represent individual participants (biological replicates). For panels C and D: bar charts with error bars represent mean ± SD, with points representing individual participants (biological replicates).

We continued our analyses of monocytes in FMF by investigating the molecular mechanisms underlying their increased responsiveness (14 HC, 7 FMF patients). We performed metabolic flux analysis by Seahorse to assess if the glycolytic metabolism and oxidative phosphorylation of monocytes is altered in FMF. Neither glycolysis nor oxidative metabolism of monocytes were affected in FMF (Figures 4A and 4B). We then calculated the extracellular acidification rate (ECAR) during basal glycolysis (after adding glucose), forced maximum glycolysis (after adding oligomycin), and the glycolytic reserve (a measure for how much glycolysis can be upregulated from the baseline; Figure 4C). There were no statistically significant changes in these parameters. Similarly, there were no changes in parameters relating to oxygen consumption rate (OCR): basal respiration, forced maximum respiration (after adding FCCP), and the respiratory reserve capacity were unaffected in FMF patients (Figure 4D). Thus, we can conclude that changes in energy metabolism dependencies are not the driver of enhanced monocyte responsiveness in FMF.

### RNA-sequencing analysis of monocytes reveals dysregulation of inflammation at baseline

We then leveraged RNA-sequencing to uncover global- and pathway-specific transcriptomic differences between the monocytes of 7 FMF-patients and 14 healthy individuals. Based on our findings thus far, we expected to observe a more pronounced inflammatory transcriptional landscape in FMF patients.

**Figure 5:**
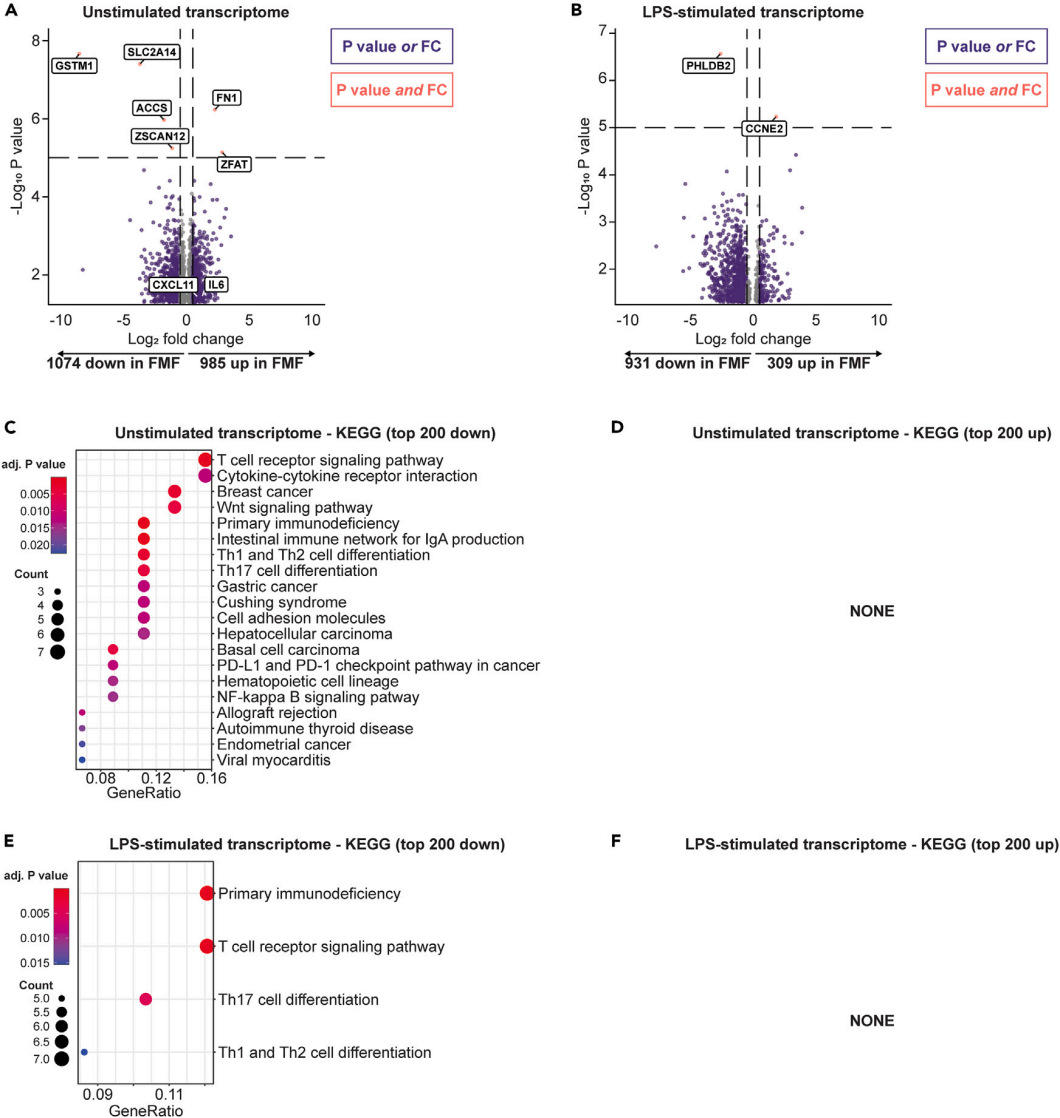
Transcriptomic analyses of monocytes in FMF (A) Volcano plot of the difference in monocyte gene expression in the absence of stimulation. The results are filtered to show genes with an unadjusted p value <0.05. (B) Volcano plot of the difference in monocyte gene expression following 4 h of stimulation with LPS. The results are filtered to show genes with an unadjusted p value <0.05. (C) KEGG enrichment among the top 200 genes that are downregulated in FMF, without stimulation. (D) KEGG enrichment among the top 200 genes that are downregulated in FMF, after 4 h stimulation with LPS. (E) There were no enriched KEGG pathways among the top 200 upregulated genes in FMF in absence of stimulation. (F) There were no enriched KEGG pathways among the top 200 upregulated genes in FMF after LPS stimulation.

We first investigated the transcriptome of unstimulated monocytes. Most FMF patients grouped together in an initial principal component analysis (PCA), although the separation from healthy controls was subtle (Figure S2A). We next performed differential expression analysis. Compared to healthy individuals, slightly more genes were downregulated in FMF patients (1074) than upregulated (985), with an unadjusted p value below 0.05 (Figure 5A). The top downregulated genes (unadjusted p value below 1 × 10^−5^) were *GSTM1* (glutathione metabolism), *SLC2A14* (a glucose transporter), *ACCS* (amino acid metabolism), and *ZSCAN* (transcriptional regulation). The top upregulated genes were *FN1* (Fibronectin 1, cell adhesion and migration) and *ZFAT* (transcriptional regulation, especially of immune genes). *IL6* and *CXCL11*, which were upregulated at the protein level, were also increased but not among the top hits. We also visualized the differentially expressed genes (Figure S2B). This revealed inter-individual heterogeneity in expression of the differentially expressed genes (DEGs), which did not prevent FMF patients and healthy controls from forming separate clusters.

After LPS stimulation, PCA revealed two subgroups of FMF patients which incompletely separated from the healthy controls (Figure S2C). 931 genes were downregulated in FMF whereas 309 were upregulated (Figure 5B). The top hit of the downregulated genes was *PHLDB2*, which enables cadherin binding activity and participates in regulation of cytoskeleton organization. The top upregulated gene was the cell-cycle protein *CCNE2*. A heatmap of the DEGs (Figure S2D) indicated that, while the two groups still clustered separately, there was a heterogeneous response to LPS in particularly the healthy control group that (also due to the relatively small sample size in this study) may have contributed to the masking of the true differential transcriptomic signature of monocytes in the FMF patients.

We performed pathway analyses on the top upregulated and downregulated genes (n = 200 in both directions). Surprisingly, in the unstimulated monocytes, KEGG enrichment analysis showed that pathways associated with immune responses were downregulated (Figure 5C), such as ‘cytokine-cytokine receptor interaction’ and ‘WNT-signaling’. Several T cell-associated pathways (such as ‘T cell receptor signaling pathway’, ‘Th17 cell differentiation’) were also downregulated, possibly due to genes that are important not only for T cells but for immune cells in general. There were no upregulated KEGG pathways among the top 200 upregulated genes (Figure 5D).

There were fewer affected KEGG pathways in the LPS-stimulated monocytes, most of them corresponding to the same general processes as in the unstimulated monocytes. Downregulated pathways included primary immunodeficiency and several T cell-associated pathways (Figure 5E). None of the KEGG pathways were enriched among the upregulated genes (Figure 5F).

Because the observed transcriptomic differences between FMF monocytes and healthy controls were subtle and the sample size did not allow for correction of p values, we performed an additional robustness analysis. We randomly divided the study participants into two groups and performed differential gene expression analysis, using the data underlying Figure 4A (unstimulated transcriptome). If the differences between FMF patients and healthy controls were due to statistical noise, the results should be similar between these randomly shuffled individuals. We observed 560 downregulated and 228 upregulated genes (Figure S3A). 15 of the upregulated genes overlapped with the results reported in Figure 4A and 8 of the downregulated genes (Figures S3B and S3C). While there were substantially fewer DEGs in this shuffled analysis, the results underline the probability of false-positives in our comparisons between FMF and healthy controls. The sequencing results of this study should therefore be interpreted with caution, and larger studies in the future are warranted.

### ATAC-sequencing reveals lower accessibility of regions near immune-regulating genes

**Figure 6:**
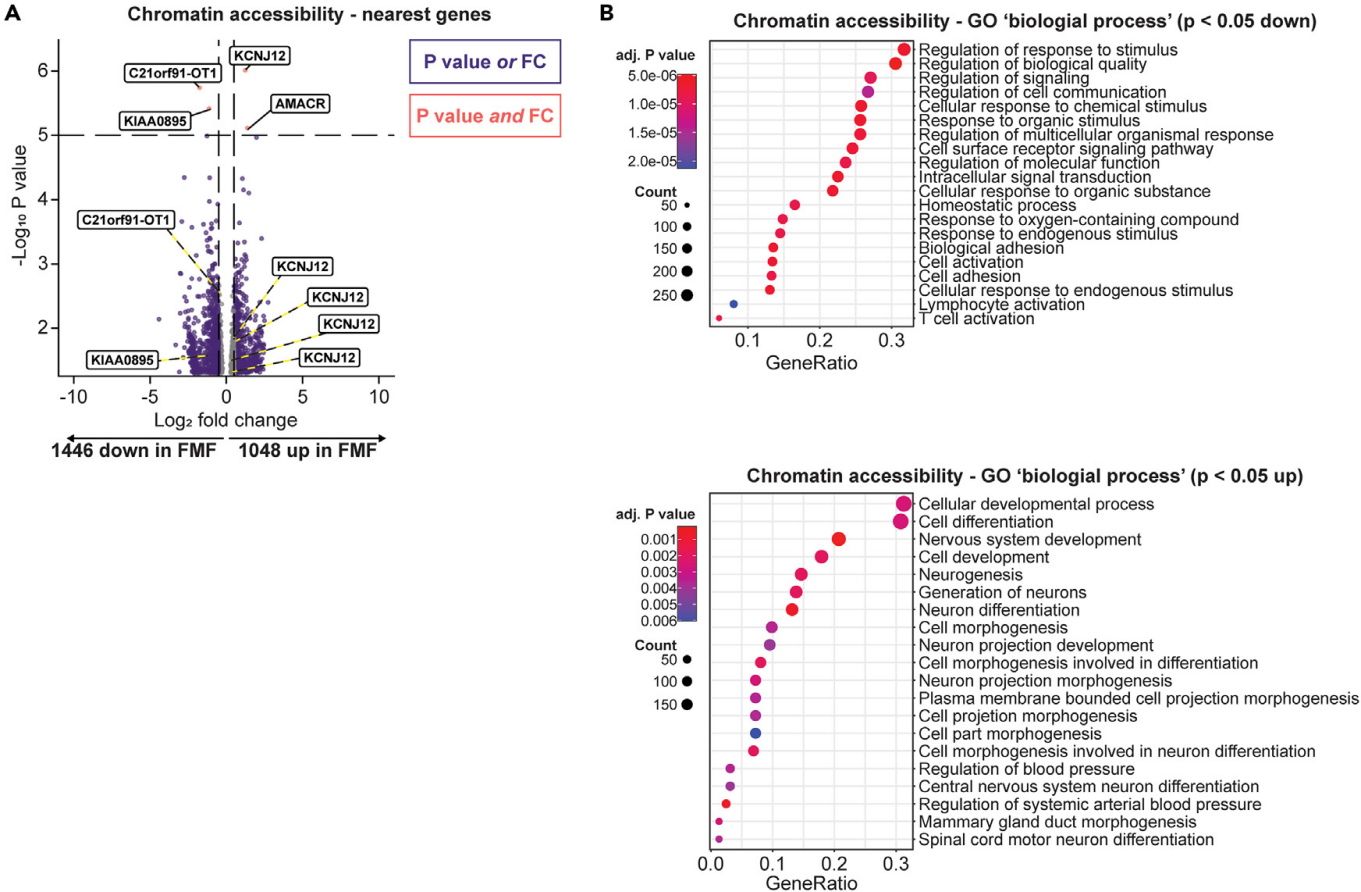
Chromatin accessibility by ATAC sequencing of monocytes in FMF (A) Volcano plot of the differences in accessible chromatin between FMF patients and healthy controls. The genomic locations that were differentially accessible are labeled as the gene they are closest to. The results are filtered to show genes with an unadjusted p value <0.05. (B) GO ‘biological process’ enrichment of the less-accessible (top panel) and more-accessible (bottom panel) regions (‘genes’).

To further investigate potential epigenetic mechanisms underlying the transcriptional and functional differences between 7 FMF patients and 14 healthy individuals, we assessed chromatin accessibility by performing ATAC-sequencing on unstimulated monocytes. We identified differentially accessible regions along the genome and paired these to the nearest gene. Similar to our transcriptomics analysis, there were more downregulated (1446) compared to upregulated (1068) loci in FMF patients (Figure 6A). The genes closest to the top downregulated loci were *C21orf91-OT1* (a lncRNA) and *KIAA0895* (a carboxypeptidase associated with microtubules). We identified a separate instance of less-accessible regions near both of these genes. The top more-accessible regions were near *KCNJ12* (a potassium channel) and *AMACR* (involved in metabolism of coenzyme A thioesters of fatty acids); especially around *KCNJ12* there were differentially accessible regions, albeit with both higher and lower accessibility.

Pathway analyses revealed that genes near less-accessible regions in FMF patients were related to regulation of the molecular response toward stimulation (Figure 6C, upper panel): ‘regulation of response to stimulus’, ‘regulation of biological quality’, and ‘regulation of signaling’ were the top GO hits (Biological process; BP). Top BP hits for genes near more accessible regions in FMF included many pathways associated with differentiation and development (Figure 6B, lower panel), as well as some pathways related to neurons (speculatively: genes potentially used in synapse formation^32^).

## Discussion

In the present study we comprehensively characterized the innate immune response of FMF patients that are medicated and in between disease flareups. In doing so, we attempted to tackle two knowledge gaps in the field. Our main goal was to describe comprehensively the innate immune responses in FMF, with a focus on myeloid cells. Secondly, we wanted to assess whether the initial genetic cause of the inflammatory phenotype of the disease leads to chronic changes in metabolic and epigenetic signatures of myeloid cells, reminiscent of trained immunity programs.^33^

We found that, compared to healthy individuals, the monocytes of FMF patients collected between disease episodes are more prone to produce cytokines upon stimulation. Especially production of IL-6 and IL-1β was increased, whereas TNF and IL-1ra were affected much less strongly. This is in line to the notion that *MEFV* mutations result mainly in the dysregulation of the inflammasome and thus IL-1β,^15^^,^^16^ and IL-6 production is strongly dependent on endogenous IL-1 release.^34^ Interestingly, IL-1ra was previously found to be unaffected in FMF,^19^^,^^20^^,^^21^ while we report here that IL-1ra is upregulated at baseline and following stimulation with *A. fumigatus*. The previous conflicting reports regarding involvement of TNF^21^^,^^22^^,^^25^^,^^26^ could be explained by differences in experimental conditions or stimuli, as we show that TNF was particularly upregulated following stimulation with Pam_3_Cys but no other stimuli. The particularly high TNF production upon Pam_3_Cys stimulation could be driven by enhanced TLR-2 expression, which has been described in FMF but should be controlled in these patients by their colchicine treatment.^35^

We also established a more encompassing inflammatory secretomic profile of monocytes in FMF by performing Olink targeted proteomics on monocyte cell culture supernatants. We noted that the monocytes of these patients produce more cytokines, chemokines and MMPs, also in the absence of stimulation. A broad trend toward upregulation of inflammatory proteins in the plasma, including those produced by monocytes *ex vivo*, suggested that these patients have low-grade chronic inflammation, despite their medication and absence of symptomatic disease.

An earlier study on another periodic fever syndrome, hyper-IgD syndrome (mevalonate kinase deficiency), showed that metabolic and epigenetic reprogramming reminiscent of induction of trained immunity is driving the hyperinflammatory profile.^36^ We therefore speculated that the initial genetic effect leading to chronic inflammation may result in metabolic and epigenetic consequences that would amplify inflammation through a trained immunity-like mechanism. However, we did not observe any shifts in monocyte central metabolism such as glycolysis or oxidative phosphorylation that have shown to characterize trained monocytes.^37^ Subsequently, analysis of the FMF monocyte transcriptome at baseline suggests that genes which are involved in immune-related pathways were downregulated, while the slightly lower number of upregulated genes did not appear to be enriched for specific pathways. Upon stimulation with LPS, the transcriptomic differences between monocytes of healthy individuals and FMF patients were less clear, and more differentially expressed genes were downregulated. This suggests that FMF monocytes display a counter-balancing tolerance profile, secondary to the genetically induced IL-1/IL-6 upregulation, rather than a trained immunity phenotype. This hypothesis is supported by chromatin accessibility data assessed by ATAC-sequencing showing many more genes being less accessible in FMF monocytes compared to controls. The less-accessible regions were around genes involved in pathways that have to do with regulation of cellular processes, which could account for the dysregulation of inflammatory signals observed in the transcriptomic- and proteomic data. However, in light of the additional robustness analyses performed, the sequencing results of this study should be interpreted with caution and follow-up research is needed to confirm our results.

Subsequently, we also assessed neutrophil function in FMF patients. While a previous study found that neutrophils of FMF patients upregulate their expression of *IL8* both at baseline and after LPS stimulation,^38^ we did not find strong evidence that this is the case at the protein level. None of the main biological functions of neutrophils were increased in FMF. If anything, we observed a slight trend toward downregulated ROS responses in the neutrophils of our cohort of FMF patients, again arguing to a counter-regulatory tolerance program. However, it should be noted that other studies found that increased secretion of important inflammatory mediators such as IL-18, S100A12, and caspase-1 is dependent on the genotype.^39^ Future studies with larger sample size and more extensive probing of neutrophil function are required to elucidate their exact phenotype in FMF.

In conclusion, we provide a comprehensive overview of monocyte (and, to a far lesser extent, neutrophil) inflammatory responses in treated FMF patients between disease attacks. We added to the evidence that FMF patients display chronic low-grade systemic inflammation and found clues that several upregulated MMPs and chemokines in the plasma are dysregulated in the disease. The metabolic and epigenetic data obtained argue against the possibility that monocytes of FMF patients acquire a long-term phenotype of trained immunity. Rather, they display an epigenetic and transcriptional counter-regulatory profile more reminiscent to induction of tolerance secondary to the chronic inflammation due to the genetic mutations in *MEFV* leading to IL-1/IL-6 dysregulation. However, we cannot exclude that the anti-inflammatory medication received by the patients may obscure a trained immunity phenotype. Follow-up research is warranted in newly diagnosed FMF patients before the start of anti-inflammatory medication, in order to fully characterize the inflammatory phenotype in this important autoinflammatory syndrome.

### Limitations of the study

There are also a few limitations to the study. The most important limitation lies in the numbers and characteristics of the participants. The low sample size prevented appropriate correction for multiple comparisons, from which the sequencing results in particular may have suffered due to the subtlety of the differences. The FMF patients studied here were all using anti-inflammatory medication, and we cannot exclude that some of the changes observed are due to medication. However, the medication is highly unlikely to explain the increased cytokine production: in contrast, it may have partially masked even stronger differences between the FMF patients and controls. Additionally, one could argue that studying medicated FMF patients is more representative of patients encountered by clinicians after their initial diagnosis. A second limitation lies in the population characteristics. Most FMF patients were women, and they were on average older than the healthy controls (Table S1; Figure 1A). Furthermore, the patients were mostly of Turkish descent, whereas our control participants were almost all of Dutch descent. It cannot be excluded that some of the differences observed here are due to the heterogeneity of the study population. However, most of our observations were in line with our expectations based on the FMF literature. It is therefore unlikely that the observed differences are completely unrelated to the disease status of the participants. Future research in a larger cohort, preferably of newly diagnosed FMF patients that have not yet started treatment, should be conducted in a way that allows for correction of these factors, or by performing comparisons with patients with a similar genetic background suffering from a different inflammatory disease.

## STAR★Methods

### Key resources table

**Table:** 

REAGENT or RESOURCE	SOURCE	IDENTIFIER
**Deposited data**		

RNA-seq data FMF and healthy controls	EGA	EGAS00001007165
ATAC-seq data FMF and healthy controls	EGA	EGAS00001007166

**Other**		

*E. coli* LPS, Further purified to specifically activate TLR-4;^58^ used at 10 ng/ml	Prepared in-house Hirschfeld et al. ^58^	-
*S. aureus*, heat-killed; used at 1 x 10^6^/ml	Prepared in-house Li et al. ^59^	-
*C. albicans*, heat-killed; used at 1 x 10^6^/ml	Prepared in-house Li et al. ^59^	-
*A. fumigatus*, heat-killed; used at 10 x 10^6^/ml (in 10% human pooled serum)	Prepared in-house Li et al. ^59^	-
Palmitic acid (C16) ; used at 400 μM	Prepared in-house Li et al. ^59^	-
MSU; used at 10 mg/ml	Prepared in-house Li et al. ^59^	-
Pam-3-Cys; used at 10 μg/ml	EMC Microcollections	L2000
PMA; used at 100 nM	Sigma	P1585
Zymosan; used at 1mg/ml	Sigma	Z4250

### Resource availability

#### Lead contact

Further information and requests for resources and reagents should be directed to and will be fulfilled by the lead contact, Mihai Netea (mihai.netea@radboudumc.nl).

#### Materials availability

This study did not generate new unique reagents.

#### Data and code availability

• Data: RNA- and ATAC sequencing data is available in the European Genome-Phenome archive under EGA IDs EGAS00001007165 and EGAS00001007166, respectively, and will be publicly available as of the date of publication. These accession numbers are also reported in the key resources table. Olink targeted proteomics data will be attached as a supplemental excel file.
• Code: The use of publicly available software packages is described in detail below. This paper does not report original code.
• Other data will be shared by the lead contact upon reasonable request.
• Any additional information required to reanalyze the data reported in this paper is available from the lead contact upon request.

### Experimental model and study participant details

#### Human participants

Blood from healthy volunteers and FMF patients was obtained for research purposes after obtaining informed consent during routine outpatient visits (Arnhem-Nijmegen Ethical Committee number NL32357.091.10; www.toetsingonline.nl/to/ccmo_search.nsf). Age, sex, and ethnicities are described in Table S1. All obtained blood was anticoagulated with EDTA and processed as soon as possible after venipuncture.

### Method details

#### Processing of EDTA whole blood

Plasma was collected and stored at -80°C after centrifugating EDTA whole blood for 10 minutes at 2970 x *g*, RT.

PBMCs were obtained from the interphase layer after density-gradient centrifugation of whole blood (diluted 1:1 with Ca/Mg-free PBS) over Ficoll-paque; 615 x *g*, 30 minutes, RT, no brakes. The PBMCs were washed three times with cold PBS and counted by a Sysmex XN-450 apparatus. Immediately afterwards, monocytes were isolated using negative magnetic selection (Miltenyi; MACS pan monocyte selection kit, human).

Neutrophils were collected from the remaining granulocyte/erythrocyte pellet that remained after PBMC isolation. To that end, the pellet was resuspended in hypotonic lysis buffer (10 mM KHCO_3_, 155 mM NH_4_Cl, in sterile water) for 10 minutes on ice. After this step to lyse erythrocytes, the neutrophils were washed with cold PBS and counted on a Sysmex XN-450 apparatus. Neutrophil isolation failed for 1 healthy individual who was subsequently excluded from neutrophil analyses, but not from all other analyses. For neutrophil data, 13 healthy controls are thus compared against 7 FMF patients.

All cell culture experiments (unless otherwise indicated) were performed in RPMI-1640 medium with Dutch modifications (Invitrogen), further supplemented with 50 mg/ml gentamicin (Centrafarm), 2 mM GlutaMAX (Gibco) and 1 mM pyruvate (Gibco). Cells were cultured at 37°C, 5% CO_2_ in a humidified incubator.

#### Seahorse metabolic flux analysis (monocytes only)

1 x 10^5^ monocytes were seeded in a Seahorse flat-bottom cell culture plate and allowed to adhere for 1 hour at 37°C (5% CO_2_). The cells were washed and incubated with Seahorse assay medium for 1 hour at 37°C (ambient CO_2_ levels). Oxygen consumption rate and extracellular acidification rate were determined as proxies for oxidative phosphorylation and glycolysis using the Seahorse XF Mitochondrial Stress Test kit and Seahorse XF Glycolysis Stress Test kit (Agilent; per the instructions of the manufacturer). Mann-Whitney U tests were used to compare healthy controls to FMF patients.

#### Reactive oxygen species measurements (neutrophils only)

2x10^5^ neutrophils (200 μl of a suspension containing 10^6^ cells/ml) were added per well in a white-plastic 96-well plate; the wells were previously filled with HBSS containing luminol (final concentration after adding cells: 0.08 mM) and the stimulus (see key resources table). The emission at 425 nm was measured using a BioTek Synergy HT apparatus. Area under the curve was used to approximate total ROS production during 1 hour of measurement. Mann-Whitney U tests were used to compare healthy controls to FMF patients.

#### Stimulation experiments (monocytes and neutrophils)

5 x 10^4^ monocytes or 5 x 10^6^ neutrophils were added to a flat-bottom 96-well cell culture plate. The cells were immediately incubated with the stimuli detailed in the key resources table for 24 hours (monocytes) or 4 hours (neutrophils). Supernatants were collected and stored at -20°C for further analysis.

#### Cytokine measurements by ELISA

The cytokines TNF, IL-6, IL-1β, and IL-1ra were measured in cell culture supernatants using DuoSet ELISA kits from R&D labs, according to the instructions provided by the manufacturer. The standard curves were used to interpolate cytokine concentrations with a combination of Gen5 software (BioTek) and Microsoft Excel. Mann-Whitney U tests were used to compare healthy controls to FMF patients.

#### Olink targeted proteomics

Plasma and cell culture supernatants were sent to Olink (Uppsala, Sweden) for targeted proteomics analysis using proprietary proximity extension assays. 92 proteins were measured (‘inflammation panel’). The (quality controlled) data was subjected to differential expression analysis in R with a linear regression approach using the Limma package.^40^ Volcano plots were generated using the EnhancedVolcano package.^41^

#### ATAC-sequencing

50∗10^3^ monocytes were washed with ice-cold PBS and immediately lysed according to the instructions of the Nextera DNA Library preparation kit. The samples were incubated 30 minutes at 37°C for the tagmentation reaction. The resulting tagged DNA fragments were extracted using the Qiagen MinElute PCR Purification kit and stored at -20°C.

Next, ATAC-seq libraries were prepared with Illumina Nextera primers and sequenced on NovaSeq 6000 platform with 50bp paired-end sequencing, where each sample was sequenced to approximate 60 million reads.

#### RNA-sequencing

Monocytes were stimulated with RPMI or LPS for 4 hours in 6 well flat-bottom cell culture plates. The cells were lysed in RLT buffer (Qiagen) and stored at -80°C until RNA extraction with Qiagen RNeasy kits. The RNA was subjected to rudimentary quality controls by Nanodrop analysis and sent to BGI Denmark for sequencing using the DNB-seq pipeline.

#### RNA-seq data analysis

We used STAR^42^ (v2.5.2b) to generate genome index and then Fastq files were aligned to the GRCh38 genome using with default parameter settings. Gene annotation was based on ENSEMBL. DESeq2 (v 1.30.1)^43^ was used for quality control and downstream analyses. Genes with less than 10 reads were removed. Suggestive DEGs were defined as raw *P Value* < 0.05. clusterProfiler (v3.18.1)^44^ was used to perform the enrichment analysis. Pathways with Benjamin-Hochberg corrected *P Value* < 0.05 were defined as significant. Heatmaps were made using the ComplexHeatmap package.

#### ATAC-seq data analysis

We followed ENCODE portal pipeline^45^ to pre-process the ATAC-seq data, including quality control, alignment to GRCh38 genome and peak calling. DESeq2 was used to perform downstream analysis. Peaks were mapped to the nearest genes. Differentially accessible peaks (DAPs) with raw *P Value* < 0.05 were defined as suggestively different. Then we used the mapped genes to perform the enrichment analysis as for RNA-seq data.

### Quantification and statistical analysis

Statistical tests were performed in R (version 4.1.0). Two-tailed p-values below 0.05 were considered statistically significant, unless otherwise indicated. All error bars indicate mean +/- SD. The statistical methods used to obtain parameters such as fold changes and *P* values are described together with the methods of each technique. In all comparisons all 7 FMF patients were compared to all 14 healthy participants (13 healthy controls for analyses of neutrophils; see above).

In addition to previously mentioned software packages, the following R packages were used: Tidyverse core packages (2.0.0),^46^^,^^47^ openxlsx (4.2.5.1),^48^ janitor (2.1.0),^49^ ggprism (1.0.6),^50^ rstatix (0.7.0),^51^ pzfx (0.3.0),^52^ viridis (0.6.2),^53^ ComplexHeatmap (2.12.1),^54^ VennDiagram (1.7.3),^55^ conflicted (1.2.0),^56^ circlize (0.4.15).^57^ Graphs were made in R and compiled into figures using Adobe Illustrator. Illustrations were made in Adobe Illustrator.

## Supplemental information

Document S1. Figures S1–S3 and Table S1

Data S1. Olink values acquired from monocyte supernatants; Olink_monocyte_supernatant.csv, related to Figures 2 and S1

Data S2. Olink values acquired from plasma; Olink_plasma.csv, related to Figure 1
