# Supplementary material for: Epigenetic, transcriptional, and functional characterization of myeloid cells in familial Mediterranean fever
Source: iScience. 2024 Feb 29;27(4):109356. doi: 10.1016/j.isci.2024.109356 (PMC10951896; doi:10.1016/j.isci.2024.109356)
Supplement: Document S1. Figures S1–S3 and Table S1 [file mmc1.pdf]

iScience, Volume *27*

Supplemental information

Epigenetic, transcriptional, and functional characterization of myeloid cells in familial Mediterranean fever

Rutger J. Röring, Wenchao Li, Ruiqi Liu, Mariolina Bruno, Bowen Zhang, Priya A. Debisarun, Orsolya Gaal, Medeea Badii, Viola Klück, Simone J.C.F.M. Moorlag, Frank van de Veerdonk, Yang Li, Leo A.B. Joosten, and Mihai G. Netea

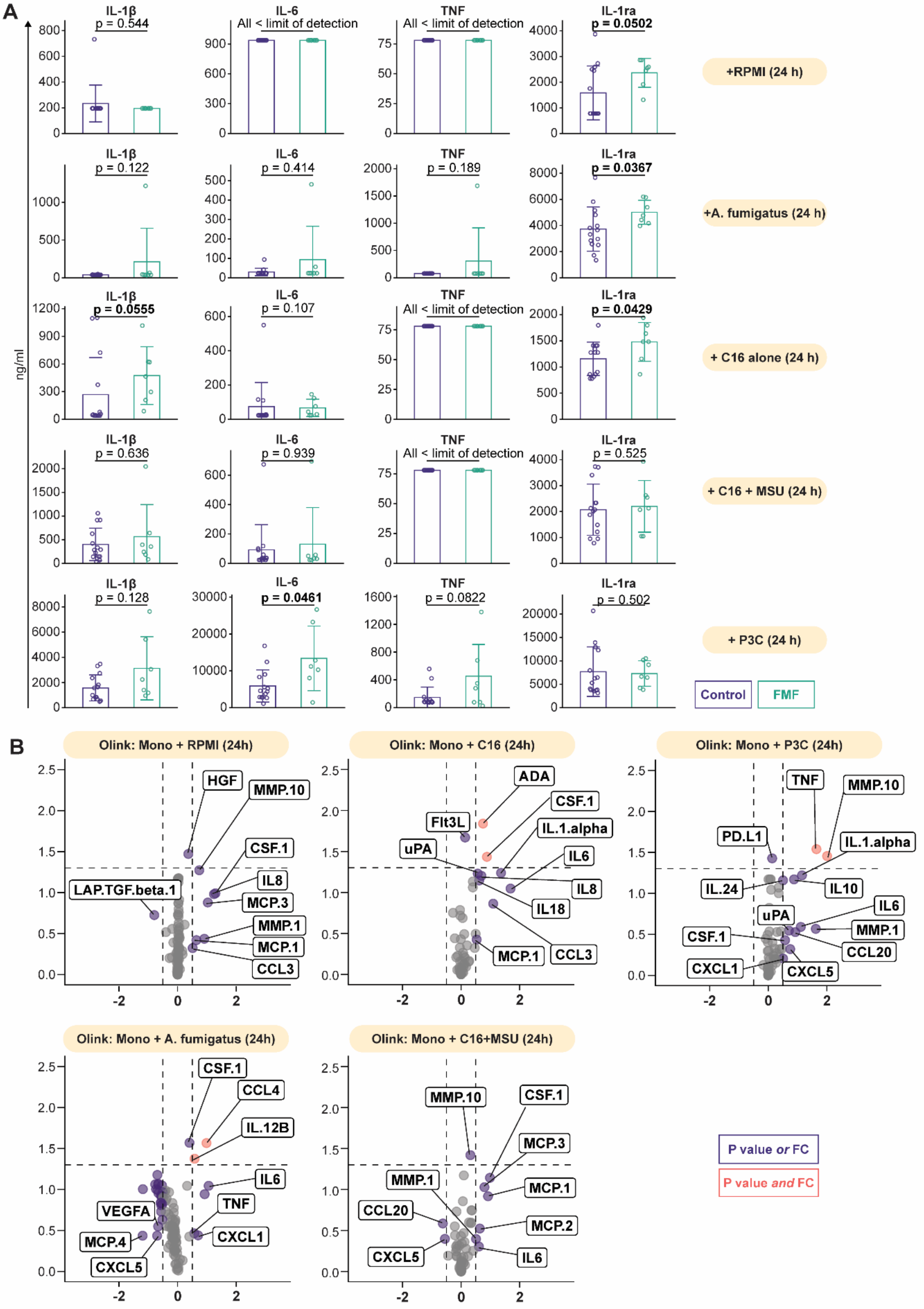

A
B
ng/ml
IL-1β
p = 0.544
IL-6
All < limit of detection
TNF
All < limit of detection
IL-1ra
p = 0.0502
+RPMI (24 h)
IL-1β
p = 0.122
IL-6
p = 0.414
TNF
p = 0.189
IL-1ra
p = 0.0367
+A. fumigatus (24 h)
IL-1β
p = 0.0555
IL-6
p = 0.107
TNF
All < limit of detection
IL-1ra
p = 0.0429
+ C16 alone (24 h)
IL-1β
p = 0.636
IL-6
p = 0.939
TNF
All < limit of detection
IL-1ra
p = 0.525
+ C16 + MSU (24 h)
IL-1β
p = 0.128
IL-6
p = 0.0461
TNF
p = 0.0822
IL-1ra
p = 0.502
+ P3C (24 h)
Control
FMF
Olink: Mono + RPMI (24h)
HGF
MMP.10
CSF.1
IL8
LAP.TGF.beta.1
MCP.3
MMP.1
MCP.1
CCL3
Olink: Mono + C16 (24h)
ADA
CSF.1
Flt3L
IL.1.alpha
uPA
IL6
IL8
IL18
MCP.1
CCL3
Olink: Mono + P3C (24h)
TNF
MMP.10
PD.L1
IL.1.alpha
IL.24
IL10
uPA
IL6
CSF.1
MMP.1
CCL20
CXCL1
CXCL5
Olink: Mono + A. fumigatus (24h)
CSF.1
CCL4
IL.12B
IL6
TNF
VEGFA
CXCL1
MCP.4
CXCL5
Olink: Mono + C16+MSU (24h)
MMP.10
CSF.1
MCP.3
MMP.1
MCP.1
CCL20
MCP.2
CXCL5
IL6
P value or FC
P value and FC

Figure S1 Inflammatory monocyte responses [Related to Figure 2]: (A) Cytokine responses (specifically IL-1β, IL-6, TNF, IL-1ra) of monocytes stimulated with RPMI, heat-killed *A. fumigatus*, C16, C16+MSU, or Pam-3-Cys; measured by ELISA. (B) Inflammatory protein production by monocytes following stimulation with RPMI, heat-killed *A. fumigatus*, C16, C16+MSU, or Pam-3-Cys; measured by Olink proximity extension assay (fold change represents FMF patients over healthy individuals).

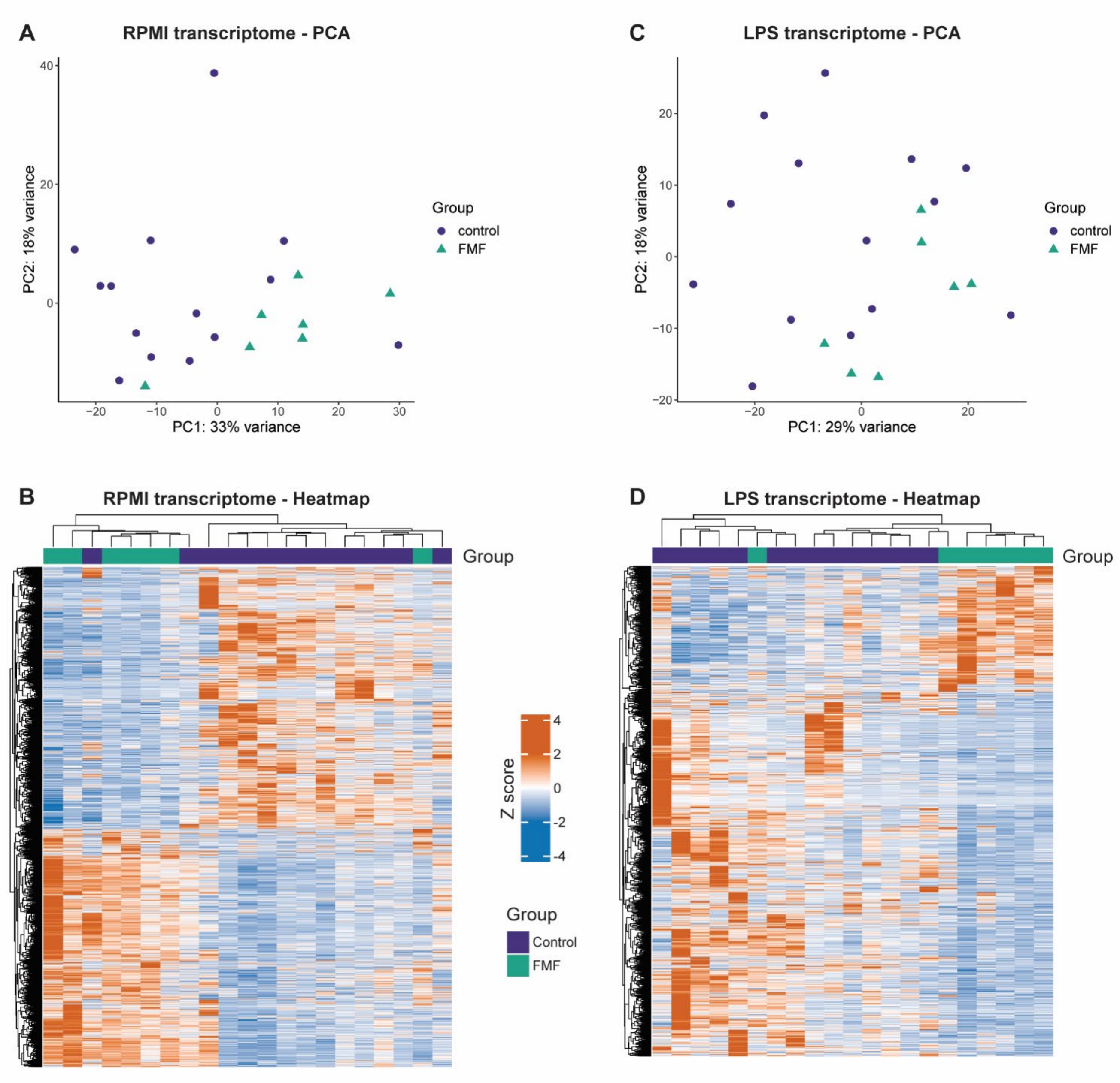

Figure S2: Transcriptomic analysis of FMF patients versus healthy controls [Related to Figure 5]. (A) Initial PCA of the unstimulated monocyte transcriptome. (B) Heatmap visualization and clustering of the suggestive monocyte DEGs (unadjusted $p < 0.05$) in the absence of stimulation. (C) Initial PCA of the LPS-stimulated monocyte transcriptome. (D) Heatmap visualization and clustering of the suggestive monocyte DEGs (unadjusted $p < 0.05$) following LPS stimulation.

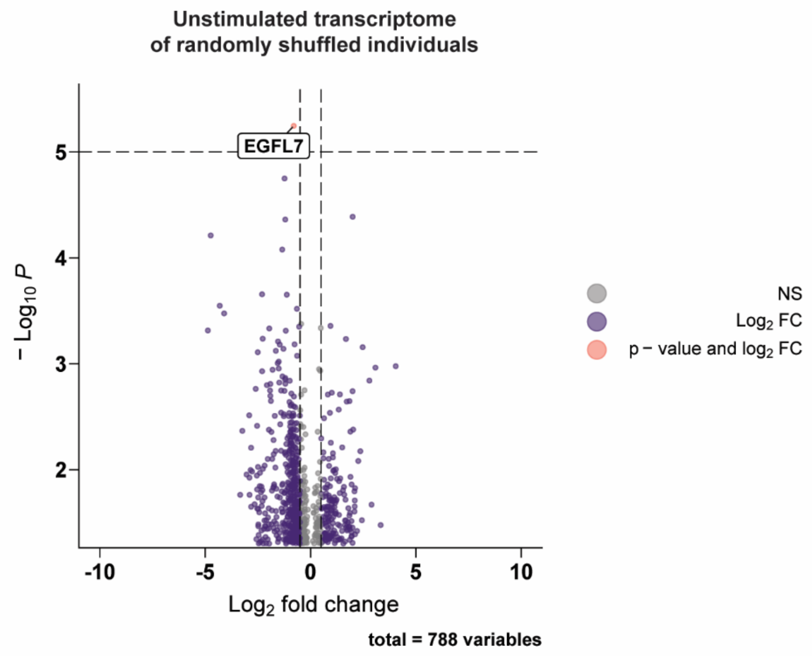

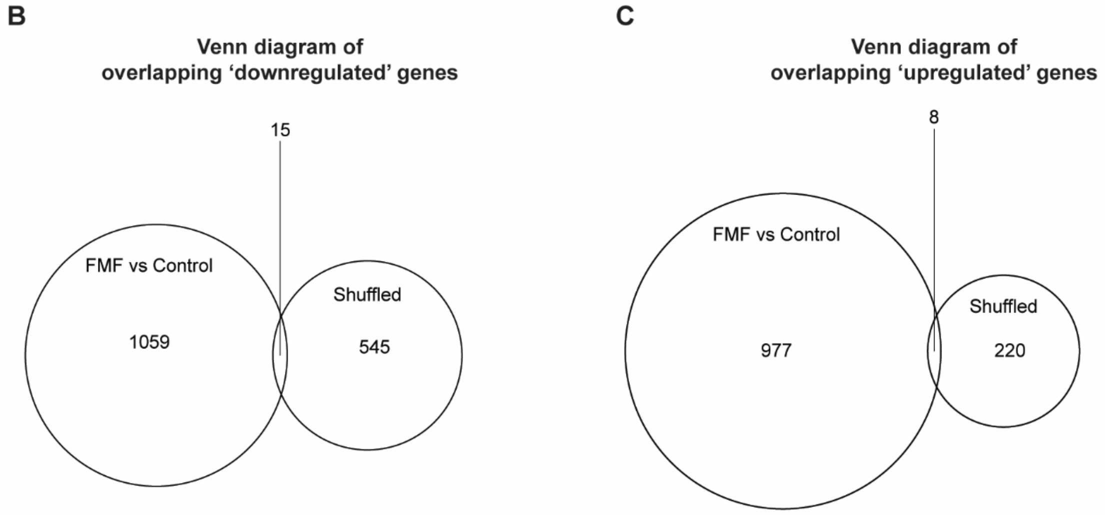

Figure S3: Analysis of robustness through shuffling of study participants [Related to Figure 5]. Individuals were randomly divided into two groups and their unstimulated transcriptome was compared. (A) Volcano plot of suggestive DEGs (unadjusted $p < 0.05$). (B) Overlap in downregulated genes between the shuffled and true comparisons. (C) Overlap in upregulated genes between the shuffled and true comparisons.

Table S1: Participant characteristics

	Control (n = 14)	FMF (n = 7)
**Age, mean (SD)**	36.36 (13.56)	46.14 (6.59)
**Sex, n female (%)**	7 (50%)	6 (85.7%)
**Ethnicity**	12 Dutch, 1 Italian, 1 German	5 Turkish, 1 Moroccan, 1 not recorded
